# Application of Antimicrobial Nanoparticles in Dentistry

**DOI:** 10.3390/molecules24061033

**Published:** 2019-03-15

**Authors:** Wenjing Song, Shaohua Ge

**Affiliations:** 1 Shandong Provincial Key Laboratory of Oral Tissue Regeneration, School of Stomatology, Shandong University, Jinan 250012, China; wenjing9207@163.com; 2 Department of Periodontology, School of Stomatology, Shandong University, Jinan 250012, China

**Keywords:** application, antimicrobial, nanoparticles, dentistry

## Abstract

Oral cavity incessantly encounters a plethora of microorganisms. Plaque biofilm—a major cause of caries, periodontitis and other dental diseases—is a complex community of bacteria or fungi that causes infection by protecting pathogenic microorganisms from external drug agents and escaping the host defense mechanisms. Antimicrobial nanoparticles are promising because of several advantages such as ultra-small sizes, large surface-area-to-mass ratio and special physical and chemical properties. To better summarize explorations of antimicrobial nanoparticles and provide directions for future studies, we present the following critical review. The keywords “nanoparticle,” “anti-infective or antibacterial or antimicrobial” and “dentistry” were retrieved from Pubmed, Scopus, Embase and Web of Science databases in the last five years. A total of 172 articles met the requirements were included and discussed in this review. The results show that superior antibacterial properties of nanoparticle biomaterials bring broad prospects in the oral field. This review presents the development, applications and underneath mechanisms of antibacterial nanoparticles in dentistry including restorative dentistry, endodontics, implantology, orthodontics, dental prostheses and periodontal field.

## 1. Introduction

The oral cavity constantly encounters a plethora of microorganisms [1]. Plaque biofilm—a major cause of caries, periodontitis and other dental diseases—is a complex community of bacteria or fungi that causes infection by protecting pathogenic microorganisms from external drug agents and escaping the host defense mechanisms [2]. Although significant numbers of study that focus on developing antimicrobial agents to overcome this problem exist, most of these attempts failed to achieve desired outcomes due to the rapid degradation and fast release of antibacterial agents causing low efficiency and safety concerns [3,4].

Nanomaterials usually refer to tiny solid particles with a diameter of 1–100 nm. Nanomaterials are promising in antibacterial therapies because of their enhanced and unique physicochemical properties such as ultra-small sizes, large surface-area-to-mass ratio and increased chemical reactivity [3]. Nanoparticles (NPs) may provide a new strategy for treating and preventing dental infections [5]. The large surface area and high charge density of NPs enable them to interact with the negatively-charged surface of bacterial cells to a greater extent resulting in enhanced antimicrobial activity [6]. Moreover, NPs combined with polymers or coated onto biomaterial surfaces was found to exhibit superior antimicrobial properties in the oral cavity [3].

Metal and organic NPs have been applied in several areas of dentistry because of their broad-spectrum bactericidal properties [5]. Smaller NPs could release their corresponding ions more to obtain a better antibacterial effect. Many kinds of research focused on the antibacterial properties of NPs and showed that NPs possessed superior antibacterial activity in bacteria of drug resistance [1,2,4]. Thus, the application of nanoparticles in dentistry might be particularly advantageous.

**Figure 1:**
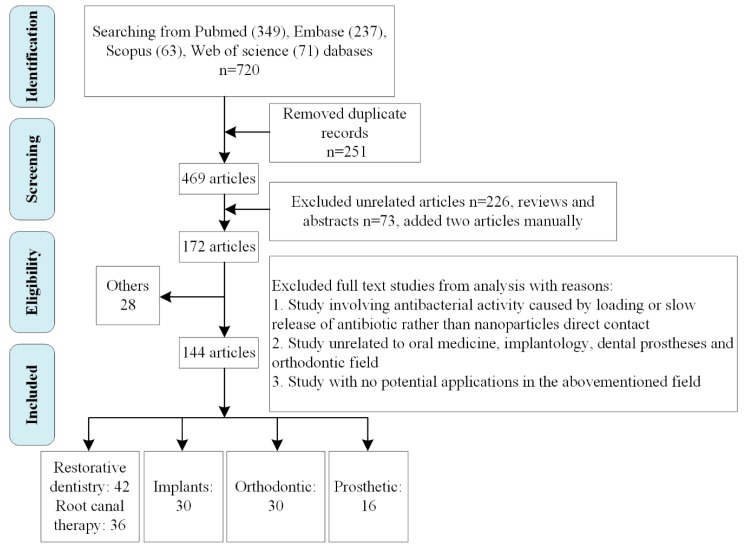
Inclusion/exclusion criteria and study flow for the systematic review.

This paper aims to present a comprehensive review (Figure 1) on the development and application of antibacterial NPs in dentistry including restorative dentistry, endodontics, implantology, dental prostheses, orthodontics and periodontal field.

## 2. Antimicrobial Applications in Dentistry

### 2.1. Antimicrobial Activity in Oral Medicine

#### 2.1.1. Restorative Dentistry

Dental caries is one of the most common infectious diseases in the oral cavity, which is usually repaired with materials of which the color is similar to that of the teeth. However, the failure of restoration or the formation of secondary caries can occur because of the lacking of antibacterial properties and demineralization caused by microorganisms and microbial acid production [1].

The development of nanotechnology has arisen the interests of many researchers. There are two methodologies to resist dental caries. The first method is incorporating inorganic antibacterial NPs into resin composites and agents to reduce microorganism biofilm with direct contact [7,8,9,10,11,12,13,14,15,16,17]. Composite resins containing 1% silver nanoparticles (AgNPs) or zinc oxide nanoparticles (ZnO NPs) exhibited a better antibacterial activity. Note that the antibacterial effect of composite resin containing ZnO NPs on *Streptococcus mutans* (*S. mutans*) was significantly higher than that containing AgNPs [1,18,19]. Additionally, *S. mutans* activity could be significantly inhibited by AgNPs which was formed in situ via a photoreduction mechanism concomitant to the polymerization reaction [3]. Even dental resins containing a low concentration of novel nanofillers possessed adequate and long-term antimicrobial properties [4]. An amount of 1 wt% quaternized copolymer functionalized nanodiamond-reinforced resin composites effectively inhibited the formation of biofilm without cytotoxicity [6]. However, some researches related to the resin luting cements with AgNPs addition and dental sealants modified with nylon-6 and chitosan nanofibers did not show an antibacterial effect against *S. mutans* [5,20]. The cooperation of graphite oxide, AgNPs and phthalocyanine molecules promoted lasting disinfection in the presence of near-infrared irradiation [21]. The form of colloidal metal oxide NPs was also proven to have superior antibacterial activity [22].

Glass ionomer cements (GICs) with a good fluoride-ion release function have been applied to prevent and reduce the occurrence of secondary caries [23]. The copper-doped glass ionomer-based materials greatly enhanced their antibacterial properties and reduced collagen degradation [24]. The addition of titanium dioxide (TiO_2_) NPs significantly improved mechanical and antibacterial activity [25]. Hexametaphosphate NPs incorporated in GICs effectively improved antibacterial properties and enhanced fluoride ion release [26]. Nevertheless, ZnO NPs as an additive into GICs could not promote the antimicrobial activity against *S. mutans* [27].

The second methodology is the usage of organic NPs to reduce demineralization and achieve remineralization. The nanoparticles of amorphous calcium phosphate (NACP) combined with polymerizable quaternary ammonium methacrylates (QAMs), such as quaternary ammonium polyethyleneimine (QPEI) [28], quaternary ammonium dimethacrylate (QADM) [29], dimethylaminohexadecyl methacrylate (DMAHDM) [7,30,31,32,33,34,35] and organic antibacterial NPs [36,37,38,39] was researched. Modified composite incorporating QPEI NPs had excellent antibacterial activity and long-term durability [28]. A composite containing both QADM and AgNPs possessed a stronger antibacterial capability, which lasts for 12 months of water-aging [29]. Antibacterial bonding agents containing DMADDM and AgNPs greatly inhibited biofilm activities such as reducing the metabolic activity, colony forming unit (CFU) and lactic acid of microcosm biofilms, even when the dental adhesive was pre-coated with salivary pellicles [36].

The combination of DMAHDM, 2-methacryloyloxyethyl phosphorylcholine (MPC) and NACP was also researched [29,30,31,32,40,41,42]. MPC, one of the most common biocompatible and hydrophilic biomedical polymers, was incorporated into dentin bonding agents and composites due to its hydrophilicity that prevents the adsorption of proteins [32,33]. NACP could release a high level of Ca and P ions, neutralize acids, and inhibit dental caries by matching mechanical properties when containing into resin composites [30,31,32].

A new rechargeable NACP composite with multiple re-release capability was developed for long-term caries inhibition [33]. The methodologies of using NACP, MPC and DMAHDM may be applicable to other dental composites, adhesives and cements to reduce the formation of plaque biofilm in restorative dentistry.

#### 2.1.2. Root Canal Therapy

Long-term or secondary dental caries may lead to pulpitis and apical periodontitis which are mainly caused by bacteria and their products. *Enterococcus faecalis* (*E. faecalis*) is mainly responsible for reinfections after root canal therapy [43,44,45]. Complex root canal anatomy makes the complete microbial cleanup particularly difficult, even with a thorough root canal preparation and filling [46,47].

The application of NPs has attracted researchers’ attention [43,48,49,50,51]. Biosynthesized AgNPs had an antimicrobial ability against *E. faecalis* [52,53,54]. Afkhami et al. reported that irrigant with 100 ppm AgNPs solution had better antimicrobial efficacy than 2.5% sodium hypochlorite (NaOCl) [55]. Poly (vinyl alcohol)-coated AgNPs (AgNPs-PVA) and farnesol (FAR) were not only more favorable for tissue repair and but also less cytotoxic in comparison with NaOCl [56]. Compared to 5.25% NaOCl, nano-MgO (5 mg/L) and chitosan NPs exhibited statistically significant long-term efficiency in the elimination of *E. faecalis* in the root canal system [57,58]. A previous study showed that the biomimetic iron oxide NPs with peroxidase-like activity enhanced antibacterial activity on root canal surfaces and in dentinal tubules [59].

The form of NPs may influence the antibacterial properties. Chlorhexidine (CHX)-AgNPs containing lyotropic liquid crystals (LLC) exhibited excellent and sustained sterilization and inhibitory effect on *E. faecalis* lasting for more than one month with a bacterial inactivation rate of ≥98.5%. Besides, no toxicity was observed in the cytotoxicity evaluation [60]. However, the latest literature showed that AgNPs irrigant was less effective against *E. faecalis* biofilm and infected dentinal tubules than NaOCl [61], which was consistent with the result that AgNPs gel was more effective as irrigant agent than solution form [62]. Moreover, NaOCl has been the most efficient irrigating solution in root canal treatment [63].

It is essential to develop antibacterial root filling materials for endodontic treatment to prevent secondary infection [64,65,66]. The addition of 0.15% AgNPs and 2.5% DMAHDM did not adversely affect the physical properties of the AH Plus paste, furthermore, the paste with nano-fillers exhibited significantly higher antibacterial activity against *E. faecalis* [67]. Calcium hydroxide (Ca(OH)_2_) is routinely used as an intracanal medicament in clinical practice. Incorporating chitosan NPs into a Ca(OH)_2_-based paste had the potential of increasing its antibacterial ability [68]. Both the nanoforms of Ca(OH)_2_ and chitosan showed superior penetration into the dentinal tubules and appreciable antibacterial efficacy [69,70,71].

The addition of NPs promoted antibacterial activity in Ca(OH)_2_ [72,73] and other filling materials [74,75,76,77]. Calcium silicate cement such as mineral trioxide aggregate (MTA) and Portland cement (PC) showed antimicrobial effects, and their antimicrobial activity was significantly enhanced by mixing them with different concentrations of AgNPs [75,78]. Similarly, the addition of QPEI NPs also reduced bacteria viability and promoted cell death [48,76,79].

### 2.2. Implants Modified with Antibacterial Nanoparticles

A dental implant is one of the most common and recognized ways to repair missing teeth. Titanium (Ti) implants are widely used in dentistry, for the high strength, durability and biocompatibility [80]. The failure of implantation is caused by the accumulation of plaque biofilm in the oral cavity during the early and healing stage. Implant infections usually include peri-implant mucositis and peri-implantitis, with incidences increasing dramatically [80,81]. The pathogenesis of dental peri-implantitis is similar to periodontitis, which is characterized by a high prevalence of Gram-negative anaerobes [81].

Among the sources of infections, *Staphylococcus aureus* (*S. aureus*), *S. mutans* and *Escherichia coli* were the principal bacterial strains to be found and tested [82,83,84]. Pathogenic bacteria/fungi such as *Porphyromonas gingivalis* (*Pg*) [85,86,87], *Aggregatibacter actinomycetemcomitansand* (*Aa*) [88], *Candida albicans* (*C. albicans*) [89] were also used as targets in a model of simulated implant infection. It is crucial to develop alternative implant materials with antibacterial ability to prevent and reduce bacterial-associated implant failure [80].

Different antimicrobial NPs were developed such as Ag [89,90,91,92,93,94,95,96], copper (Cu) [95], ZnO [87,97,98], titanium dioxide (TiO_2_) [99,100,101] and selenium (Se) [102]. AgNPs showed significant antimicrobial activity against Gram-negative and Gram-positive bacteria [86,103]. Ti surface loaded with 0.05 ppm AgNPs was sufficient to inhibit Gram-positive and Gram-negative species, and the latter was more susceptible to AgNPs. However, the NPs applied in this study exhibited cytotoxicity on osteoblasts, thus limited its clinical application [91]. Ag plasma immersion ion implantation-treated Ti surface showed a higher inhibitory effect on *Fusobacterium nucleatum* (*Fn*) than *S. aureus* [94].

Ti substrates combination with hydroxyapatite [103] or chitosan NPs [85,104], surface modification of Ti-implants [83,87,105,106,107,108] and application of composite coating [82,104,109] exhibited superior antibacterial activity and better biocompatibility. Zhong et al. [104] prepared a phase-transited lysozyme (PTL)-hyaluronic acid-chitosan/nano-Ag composite coating on Ti surface by the layer-by-layer self-assembly method. At the first four days, the inhibition rate against *S. aureus* was close to 100% and kept in the range of 65–90% after two weeks. Therefore, Ti modified with coatings could keep a strong and stable antibacterial activity for a long time.

TiO_2_ nano-array modified Ti substrate prepared by our group was found to promote the adhesion, proliferation and osteogenic differentiation of human periodontal ligament stem cells. In addition, TiO_2_ nanorod arrays (TNRs) exhibited superior antifungal/antibacterial properties [105,106]. The preliminary results showed that surface-area-to-mass ratio, roughness and hydrophilicity were improved and enhanced after modification with TNRs [105]. More importantly, TNRs presented significantly higher antifungal (*C. albicans*) and antibacterial (*Aa* and *Pg*) activity toward both biofilm and planktonic states than pure Ti after ultraviolet (UV) irradiation [106]. Cu [83,110], ZnO [87,97,98] and Se [102] also imparted certain antibacterial properties to implanting materials.

At present, most of the studies have been carried out in vitro or in vivo. The effect of nanoparticle-modified implants on human micro-environment and the size, loading doses as well as bio-safety of various NPs have yet to be further investigated.

### 2.3. Orthodontics

Fixed orthodontic appliances/treatments are inclined to plaque biofilm accumulation and enhance the chance of enamel demineralization (also called white spot lesions, WSLs), which is the initial performance of dental caries due to organic acid produced by the biofilm of microorganisms [111,112]. Though oral hygiene education and mechanical therapy can prevent and remove the plaque biofilm, more effective methods should be developed to prevent WSLs with long-term anti-adhesion and antibacterial properties independent of patient’s cooperation [111,113].

Studies showed that the addition of antimicrobial NPs to orthodontic adhesive agents [111,114,115,116,117,118,119,120,121,122] and resin-modified glass ionomers cements (RMGICs) [112,121,122,123] might prevent plaque accumulation and bacterial adhesion. Sodagar et al. showed that all experimental groups reduced the viable bacterial count by comparing to the control group, 5% Ag/hydroxyapatite nano-fillers had good antibacterial properties and shear bond strength [116]. The addition of CuO NPs promoted the antimicrobial property without adverse effects on shear bond strength [111]. On the contrary, the addition of TiO_2_ NPs presented better antimicrobial activity while weakening the shear bond strength [114]. RMGICs were widely used in orthodontic appliances due to their outstanding fluoride ions release [121,122]. MPC, DMAHDM, AgNPs and NACP were separately incorporated into RMGICs and all obtained optimal antibacterial results [112,121,122,123]. A novel multifunctional orthodontic cement which was developed with strong antibacterial effect could inhibit bacteria on the cement and in the vicinity away from the brackets [112,123].

Nanotechnology was also applied to orthodontic accessories such as brackets [113,124], orthodontic wires/ligatures [125,126,127,128], micro-implants [129] and orthodontic retainers [130] since biofilms are more prone to aggregate on the surfaces of irregular structures. Evidence showed that the addition of CuO NPs and (CuO-ZnO) NPs had a better antimicrobial effect and control than ZnO NPs groups [124]. Animal experiments showed that AgNPs coated brackets effectively inhibited the growth of *S. mutans* up to 45 days without cytotoxicity [113]. Nickel-Titanium (NiTi) and stainless-steel archwires modified with antibacterial NPs of Ag and ZnO exhibited excellent antibacterial activity and biocompatibility [127,128]. In the debonding stage in vivo, the addition of AgNPs into orthodontic retainers had a strong antimicrobial effect against *S. mutans* [130].

### 2.4. Other Applications

#### 2.4.1. Antimicrobial Application in Prosthetic Fields

Wearing removable/complete dentures for a long term is prone to microbial aggregation, which can lead to denture stomatitis [131]. Polymethyl methacrylate (PMMA) has been the most common utilized biomaterial for removable partial or complete dentures, although it exhibits relatively poor antimicrobial properties [132].

Various nanofillers have been incorporated into biomaterials to improve antibacterial activity. The addition of inorganic NPs such as Ag [133,134,135,136], platinum [137], Zn/ZnO [138], Ti/TiO_2_ [139,140,141] and zirconium oxide (ZrO_2_) [142,143] exerted excellent antibacterial effects. TiO_2_ NPs have a large spectrum of activity against microorganisms including Gram-negative and Gram-positive bacteria and fungi [140]. Additionally, TiO_2_ NPs improved the antimicrobial behavior of PMMA by significantly reducing bacterial adherence as TiO_2_ ratio increased [139]. PMMA incorporation with the nanofillers of TiO_2_ and silicon dioxide mixture had a superior antibacterial activity under UV, which could degrade microorganisms with prolonged exposure [144]. A study compared four inorganic antibacterial materials and showed that 3 wt% Ag-supported Zr phosphate (Navaron) and tetrapod-like zinc oxide whiskers (T-ZnOw) in ZrO_2_-Silanized aluminum borate whiskers (ABWs)/PMMA composites possessed substantially higher antibacterial activity and exhibited no cytotoxicity, even though the filler of TiO_2_ NPs and (Ag/TiO_2_) NPs were slightly cytotoxic [141].

Non-metallic NPs possess powerful antibacterial properties. CHX-NP-coated silicone specimens exhibited antifungal action while CHX-hexametaphosphate (HMP) showed slow, sustained antibacterial properties [145]. As organic polymers, chitosan and graphene oxide are well known for their antibacterial properties. The application of chitosan NPs effectively inhibited fungal and bacterial growth within 48 hours [131]. Graphene oxide incorporated into PMMA had long term antimicrobial-adhesive effects without compromising mechanical properties [146]. However, the black color of graphene oxide limited its clinical application.

In summary, the current NPs still have certain disadvantages. In the future, antibacterial nanomaterials with excellent mechanical and aesthetic functions are expected to be developed and applied in clinical practice.

#### 2.4.2. Periodontics and Preventive Medicine

Periodontitis is a type of bacterial infectious disease caused by microorganisms. The new composite consisted of DMAHDM, MPC and NACP effectively inhibited the recognized periodontitis-related pathogens without compromising the mechanical properties [147,148,149,150]. Studies in vitro showed that the mouthwash solution containing TiO_2_ NPs showed superior antibacterial activity against oral pathogenic microorganisms [151]. Nanoparticles of Zn [152,153], Ag [2,154,155,156,157,158,159,160] synergistic with CHX [161,162] or doxycycline [163] showed significant antibacterial effect in an in vitro subgingival biofilm mode. Hexagonal form of boron nitride (hBN)—referred as “white graphite”—showed high antibiofilm activity on preformed biofilm and exhibited no cytotoxic effect on cells at the concentration range of 0.025–0.1 mg/mL [164].

In addition to active and effective antibacterial treatment, NPs also play a major role in the field of prevent the oral diseases. Nanoparticles of sodium fluoride (NSF) fluoride-based varnishes showed expected antibacterial effects when compared to silver diamine fluoride (SDF) varnishes—the gold standard for anticariogenic agents [165,166,167]. The toothbrush impregnated with AgNPs reduced the number of the putative periodontal pathogens [168,169]. The abovementioned methods can be applied to prevent the early childhood pit and fissure caries.

At present, the primary purpose of research on periodontitis and oral prevention is to achieve the long-term sustained antibacterial effect, and researchers mainly focused on the loading and sustained release of NPs on drugs [170,171,172].

## 3. Antibacterial Mechanism

Nanoparticles are capable of attaching and penetrating cell walls of both Gram-positive and Gram-negative bacteria, which disturbs cell function by releasing related ions [4]. Therefore, NPs are advantageous for the prevention and treatment of diseases caused by drug-resistant microorganisms and inhibition of biofilm formation.

The antibacterial mechanism of NPs can be roughly divided into three types although the specific mechanism of action is not yet clear. The antibacterial mechanisms are described as follows: (1) interacting with peptidoglycan cell wall and membrane and causing cell lysis; (2) interacting with bacterial proteins and disrupting protein synthesis; (3) interacting with bacterial (cytoplasmic) DNA and preventing DNA replication [4,120,141].

As the representative of inorganic NPs, AgNPs have been reported to interact with the abovementioned structures to inhibit respiratory chain enzymes and to interfere with membrane permeability. AgNPs could convert oxygen into active oxygen by its catalytic action leading to the structural damage of the microorganisms, which is called the “oligodynamic action” of Ag [133].

As the representative of organic NPs, chitosan is a derivative of chitin, the second most abundant natural biopolymer. Chitosan is biocompatible and biodegradable, and possesses a broad range of antimicrobial activity [56]. Moreover, chitosan NPs are inferred to have a similar antibacterial mechanism as AgNPs.

Despite the significant antibacterial activity of NPs, limitations for application still exist, which include inconsistent antibacterial concentrations against micro-biofilm, toxicity and potentially undesirable effects on the human body.

## 4. Toxicity

The toxicity of NPs may be influenced by many factors. Some studies did not explore the toxicity of NPs [18,45,64,67,124], while others showed that NPs with antibacterial properties did not exhibit cytotoxicity within a certain concentration ranges [11,57,80,92,94,101,115,120,146,154,164]. Not surprisingly, low concentration NPs with antibacterial properties were non-toxic while the high-concentration ones exhibited more pronounced cytotoxicity [110], and even some researchers found that the toxicity of NPs exhibited a dose-dependent effect [6,9,159].

However, there are other sounds according to the toxicity of NPs [19,60,95,141]. A previous study found that the toxicity of NPs had a strong correlation with the time, rather than with the concentration of antimicrobial NPs [19]. A favorable outcome was that the addition of antibacterial NPs made the original materials less toxic but more biocompatible [60,84,90]. In summary, the toxicity of antimicrobial NPs is affected by a variety of factors such as dosage, types, particle size, distribution, duration of action, interaction with other components and so on.

NPs can easily enter the body and accumulate in organs leading to symptoms of poisoning due to the extremely small particle size. To date, no study has been conducted to test the cytotoxicity of NPs on human beings. Additionally, though a few pieces of research have explored the antibacterial toxicity of different NPs [50,141], there are no uniform indicators to standardize the toxicity of antibacterial NPs. As a result, it is difficult to compare the toxicity among different NPs. The toxicity of antimicrobial NPs is worthy of being explored in the same condition in dentistry.

## 5. Conclusions

In this review, applications of antibacterial NPs in dentistry are explored. The antibacterial mechanisms and bio-safety are also discussed. Our results illustrate that antimicrobial NPs have wide ranges of applications in restorative dentistry, endodontics, implantology, dental prostheses, orthodontics and other dental fields. NPs have an excellent antibacterial effect, but their antibacterial properties are affected by the concentration, type, form and other factors. The specific antibacterial mechanism and toxicity are not yet clear, and thus further research is needed to address the exact mechanism. Two limitations in the present study should be highlighted. Firstly, the applications of antimicrobial NPs in dentistry are not completely discussed in this review, as only the papers published during the last five years are included. Secondly, language bias might exist since only papers published in English are included.
